# Impact of the COVID-19 Pandemic on Peripartum Fever: A Comparative Study

**DOI:** 10.7759/cureus.106533

**Published:** 2026-04-06

**Authors:** Aliette D'Hoop, Soraya Cherifi, Eric Cavatorta, Catherine Riera

**Affiliations:** 1 Gynecology, Hôpitaux Iris Sud, Bruxelles, BEL; 2 Internal Medicine, Centre Hospitalier Universitaire (CHU) Charleroi, Charleroi, BEL; 3 Pediatric Medicine, Centre Hospitalier Universitaire (CHU) Charleroi, Charleroi, BEL; 4 Gynecology, Centre Hospitalier Interrégional Edith Cavell (CHIREC), Braine l'Alleud, BEL

**Keywords:** covid-19, covid-19 in pregnancy, maternal fever, newborn health, peripartum

## Abstract

Background: COVID-19 has been associated with febrile conditions during the peripartum period. Although understanding of COVID-19 has improved, the real impact of this disease among pyretic women in the peripartum period is poorly described. Our aim was to assess the etiology and consequences of maternal and neonatal fever since the pandemic.

Methods: This comparative retrospective study is based on a comparison of all pyretic pregnant women admitted for delivery at >15 weeks' gestation at the Centre Hospitalier Universitaire (CHU) Charleroi in Charleroi, Belgium, during the COVID-19 pandemic (February 21, 2020, to December 14, 2020) and outside the pandemic (February 21, 2019, to December 14, 2019).

Results: A total of 80 patients were included in both cohorts, 49 including four COVID-19-infected patients during the pandemic period and 31 in the control group. The main fever etiologies were intra-amniotic infection (p=0.12) and urinary tract infection (p=0.75). Pandemic newborns had better outcomes regarding neonatal intensive care unit (NICU) admission (p=0.004), fever (p=0.02), Apgar score 7 at one minute (p=0.039), and time of hospitalization (p=0.045).

Conclusion: The COVID-19 pandemic had no impact on peripartum fever etiology and maternal adverse events, but there is a potential association between improved pyretic outcomes during the COVID-19 pandemic. This positive effect might be explained by the behavioral and socio-environmental modifiers influencing health and well-being during the pandemic.

## Introduction

Intrapartum fever is defined as an elevation of body temperature to ≥38°C during delivery. According to recent studies, the incidence of intrapartum fever ranges between 6.8% and 10.1% [1,2].

Increased temperature during labor may result from infectious or non-infectious conditions. Non-infectious causes include epidural analgesia [3], delivery in an overheated room, and normal physiological changes during labor [4]. Regarding infectious causes, chorioamnionitis represents the most frequent etiology, followed by endometritis, urinary tract infections, wound infections, and sepsis [5].

Regardless of etiology, neonates are more likely to present with a low Apgar score and meconium-stained amniotic fluid and require admission to the neonatal intensive care unit (NICU). For mothers, intrapartum fever has been associated with an increased rate of cesarean delivery [6,7].

The outbreak of COVID-19 has not spared pregnant women. Among women in labor, the proportion testing positive has ranged from 7% to 27%, with the majority being asymptomatic (75-88%) [8-10]. For pregnant women in general, fever represents the most frequently reported symptom (58%) [11]. Infected pregnant women, particularly those with early detection of infection during pregnancy or with more symptomatic presentations, must be followed closely due to their higher rates of low birth weight, preterm birth, and cesarean delivery. However, severe COVID-19 infection appears to be associated with increased maternal morbidity in some studies, although serious adverse events remain relatively uncommon in infected mothers and newborns [11-13].

Maternofetal transmission remains uncertain, although vertical transmission has been confirmed in a limited number of cases [14]. When present, neonatal symptoms generally mirror those observed in the mother [15].

In this study, we investigated intrapartum fever among pregnant women during the COVID-19 pandemic. We analyzed the causes of fever, identified pathogens, assessed maternal and neonatal outcomes during the pandemic period, and compared them with those observed in a control cohort before the pandemic.

The primary objective was to evaluate the impact of the COVID-19 pandemic on the incidence and etiology of intrapartum fever. The secondary objectives were to describe the distribution of infectious pathogens and to assess associated maternal and neonatal outcomes in both periods.

Overall, our aim was to better characterize febrile pregnant women during the pandemic period, from its onset and prior to vaccine implementation, in comparison with the pre-pandemic setting.

This article was previously presented as a poster at the 25th Annual Congress of the Belgian Society of Internal Medicine on December 11, 2021.

## Materials and methods

Setting

The Centre Hospitalier Universitaire (CHU) Charleroi is a tertiary care hospital located in Belgium, where approximately 1,800 deliveries are performed annually.

Study design

This retrospective observational study compared all pregnant women presenting with intrapartum fever who were admitted for delivery at the CHU Charleroi during two periods: before the COVID-19 pandemic and during the pandemic.

The two cohorts covered the same time frame of the year and the same number of days: from February 21, 2019, to December 14, 2019, for the pre-pandemic period and from February 21, 2020, to December 14, 2020, for the pandemic period.

Data collection

Data were collected retrospectively and anonymously from hospital medical records.

Pregnant women admitted for delivery beyond 15 weeks' gestation were eligible for inclusion if they presented with intrapartum fever (≥38°C). In the pre-pandemic period, inclusion was based solely on the presence of fever, whereas in the pandemic period, only women with intrapartum fever who had a documented nasopharyngeal SARS-CoV-2 reverse transcription-polymerase chain reaction (RT-PCR) test at admission were included. 

Clinical and microbiological data were retrospectively collected from hospital records, including maternal temperature, SARS-CoV-2 test results, and blood culture findings for both mothers and neonates during the two study periods. Microbiological samples (including blood cultures and other relevant specimens) were collected prior to the initiation of antibiotic therapy, according to institutional protocols.

Maternal clinical information was collected, including demographic characteristics (age, body mass index, gravida, parity, scarred uterus, anesthesia), comorbidities, co-infections, obstetric pathologies, gestational age at delivery, mode of delivery, and identified bacterial and viral pathogens. The etiology of fever was determined retrospectively based on clinical presentation, laboratory findings, and microbiological results, according to standard clinical practice. Maternal outcomes were also analyzed. 

Neonatal outcomes included fetal distress, meconium-stained amniotic fluid, admission to the NICU, Apgar score, umbilical cord pH, identified pathogens, and length of hospital stay.

Patients infected with COVID-19 were analyzed in greater detail regarding obstetric and medical history, laboratory and bacteriological findings, chest X-ray results, treatment, clinical parameters, and hospital readmission. Clinical severity was classified according to the National Institutes of Health (NIH) guidelines [16]. 

The qualitative variables were compared using the chi-squared and exact Fisher's test. Continuous variables were compared via Student's t-test, normality test, and Mann-Whitney rank-sum test. The statistical tests were carried out with an alpha threshold set at 0.05, using Pearson's test or exact Fisher's two-tailed test depending on the context. A p-value of 0.05 was considered statistically significant. The statistical analyses were carried out using VassarStats, SigmaPlot 12.0 (Systat, Chicago, Illinois, United States), and Excel (Microsoft Corporation, Redmond, Washington, United States).

Given the retrospective design and sample size, no multivariable analysis was performed to adjust for potential confounders. However, both cohorts were defined over identical time periods to limit seasonal bias, and baseline characteristics were compared between groups.

## Results

A total of 80 women presenting with intrapartum fever during labor were included in the study (Table 1). A higher number of febrile patients were observed during the pandemic period (COVID-19 group, n=49; 3.9% of deliveries) compared with the control group (n=31; 2.1% of deliveries), although the difference was not statistically significant (p=0.07).

**Table 1:** Maternal characteristics (*)Anesthesia includes epidural, spinal, and general anesthesia. (**)Hematological disorder includes drepanocytosis, thalassemia, and von Willebrand disease. Continuous variables were compared using Student's t-test or the Mann-Whitney U test based on normality. Categorical variables were compared using the chi-squared test or Fisher's exact test when appropriate. Two-tailed p-values are reported (α=0.05). The dash ("-") indicates that no statistical test was performed for this category, either because the sample size was too small (n<5) or because the comparison between groups was not applicable.

Category	Maternal characteristics	COVID-19 group total patients: n=49 (%)	Control group total patients: n=31 (%)	Statistic test	P-value
Maternal	Mean age (IQR)	30 (24-37)	32 (26-38)	t=1.31	0.19
Maternal	Mean body mass index, kg/m^2^ (IQR)	28 (17-39)	26 (16-37)	t=1.12	0.28
Maternal	Median gravida (IQR)	2 (1-14)	2 (1-8)	-	0.24
Maternal	Mean parity (IQR)	1 (0-7)	1.02 (0-5)	t=0.09	0.96
Maternal	Nulliparous	27 (55%)	16 (52%)	χ²=0.09	0.76
Maternal	Scared uterus	4 (8%)	3 (10%)	-	0.55
Maternal	Anesthesia (*)	35 (71%)	27 (87%)	χ²=2.67	0.1
Comorbidity	Chronic hypertension	1 (2%)	0	-	1
Comorbidity	Dysthyroidism	4 (8%)	2 (6%)	-	1
Comorbidity	Diabetes	1 (2%)	0	-	1
Comorbidity	Asthma	3 (6%)	3 (10%)	-	0.67
Comorbidity	Cardiovascular disease	1 (2%)	0	-	1
Comorbidity	Anemia	1 (2%)	1 (3%)	-	1
Comorbidity	Hematological disorder (**)	1 (2%)	0	-	1
Obstetric	Dichorionic twins	1 (2%)	1 (3%)	-	1
Obstetric	Gestational anemia	2 (4%)	3 (10%)	-	0.37
Obstetric	Gestational diabetes	6 (12%)	3 (10%)	-	0.51
Obstetric	Spontaneous rupture of the membrane >18h	7 (15%)	10 (32%)	χ²=3.5	0.06
Obstetric	Preeclampsia	2 (4%)	3 (10%)	-	0.37
Infection	Hepatitis B chronic	1 (2%)	2 (6%)	-	0.55
Infection	Syphilis	2 (4%)	1 (3%)	-	1
Infection	HIV	1 (2%)	2 (6%)	-	0.55
Outcome	Number of deliveries (total over study period)	1527	1446	-	-
Outcome	Number of patients with fever during the study period	49 (3.2%)	31 (2.1%)	χ²=3.05	0.07

Maternal baseline characteristics were comparable between the two groups. Spontaneous rupture of membranes lasting more than 18 hours occurred less frequently during the pandemic period (p=0.06).

The etiology of maternal fever was identified in 59% of cases during the pandemic and in 58% of cases in the control group (Table 2). The most frequent causes were intra-amniotic infection (22% vs. 39%; p=0.12) and urinary tract infection (16% vs. 13%; p=0.75).

**Table 2:** Maternal fever etiology Continuous variables were compared using Student's t-test or the Mann-Whitney U test based on normality. Categorical variables were compared using the chi-squared test or Fisher's exact test when appropriate. Two-tailed p-values are reported (α=0.05). NA indicates data not available. The dash ("-") indicates that no statistical test was performed for this category, either because the sample size was too small (n<5) or because the comparison between groups was not applicable.

Maternal fever etiology	COVID-19 group total patients: n=49 (%)	Control group total patients: n=31 (%)	Statistic test	P-value
Intra-amniotic infection	11 (22%)	12 (39%)	χ²=2.45	0.12
Urinary tract infection	8 (16%)	4 (13%)	-	0.75
Wound infection	2 (4%)	2 (6%)	-	1
Ovarian vein thrombosis	2 (4%)	0	-	0.5
Pneumonia	4 (8%)	0	-	0.15
Influenza	1 (2%)	0	-	1
COVID-19	4 (8%)	0	-	0.15
Otorhinolaryngology	0	1 (3%)	-	0.39
Unknown	20 (41%)	13 (42%)	χ²=0.01	0.92

Among bacterial pathogens, *Escherichia coli *was the most commonly identified organism in both periods (20% vs. 32%; p=0.23) (Table 3). Four cases of SARS-CoV-2 infection (8%) were recorded during the pandemic period.

**Table 3:** Maternal microorganism infection Continuous variables were compared using Student's t-test or the Mann-Whitney U test based on normality. Categorical variables were compared using the chi-squared test or Fisher's exact test when appropriate. Two-tailed p-values are reported (α=0.05). The dash ("-") indicates that no statistical test was performed for this category, either because the sample size was too small (n<5) or because the comparison between groups was not applicable.

Type of microorganism	Microorganism	COVID-19 group total patients: n=49 (%)	Control group total patients: n=31 (%)	Statistic test	P-value
Gram-negative bacilli	Escherichia coli	10 (20%)	10 (32%)	χ²=1.42	0.23
Gram-negative bacilli	Morganella morganii	1 (2%)	0	-	1
Gram-negative bacilli	*Enterobacter cloacae *complex	0	1 (3%)	-	0.38
Gram-negative bacilli	Klebsiella oxytoca	0	1 (3%)	-	0.38
Gram-positive cocci	Enterococcus faecalis	0	2 (6%)	-	0.15
Gram-positive cocci	Coagulase-negative *Staphylococcus*	0	1 (3%)	-	0.38
Gram-positive cocci	Staphylococcus lugdunensis	2 (4%)	0	-	0.52
Gram-positive cocci	Streptococcus oralis	2 (4%)	1 (3%)	-	1
Gram-positive cocci	Streptococcus anginosus	1 (2%)	1 (3%)	-	1
Gram-positive cocci	Streptococcus bovis	0	1 (3%)	-	0.38
Gram-positive cocci	Group B *Streptococcus*	4 (8%)	2 (6%)	-	1
Gram-positive cocci	Aerococcus christensenii	0	1 (3%)	-	0.38
Others	*Ureaplasma *spp.	4 (8%)	6 (19%)	-	0.17
Others	*Gardnerella *spp.	5 (10%)	5 (16%)	-	0.5
Others	*Candida *spp.	3 (6%)	3 (10%)	-	0.67
Others	Mycoplasma hominis	0	1 (3%)	-	0.38
Others	Polymorpha	2 (4%)	4 (13%)	-	0.2

Regarding maternal outcomes, no significant differences were observed between the COVID-19 group and the control group in terms of length of hospital stay (median 4 days vs. 5 days; p=0.26), postpartum hemorrhage (27% vs. 16%; p=0.28), or intensive care unit (ICU) admission (9% vs. 13%; p=0.70).

Similarly, neonatal outcomes such as intrauterine fetal death (14% vs. 16%; p=1), preterm birth (17% vs. 19%; p=0.65), meconium-stained amniotic fluid (12% vs. 11%; p=1), and mode of delivery were comparable between the two groups (Table 4).

**Table 4:** Neonatal characteristics, obstetric outcome, and neonatal outcome (*)Fetal condition includes selective intrauterine growth restriction, macrosomia, cardiovascular defect, acute fetal syndrome, and seroconversion during pregnancy. Continuous variables were compared using Student's t-test or the Mann-Whitney U test based on normality. Categorical variables were compared using the chi-squared test or Fisher's exact test when appropriate. Two-tailed p-values are reported (α=0.05). The dash ("-") indicates that no statistical test was performed for this category, either because the sample size was too small (n<5) or because the comparison between groups was not applicable.

Category	Neonatal characteristics	COVID-19 group n/total (%)	Control group n/total (%)	Statistic test	P-value
Survival	Alive	42/49 (86%)	26/31 (84%)	-	1
Survival	Fetal death in utero	7/49 (14%)	5/31 (16%)	-	1
Birth timing	At term	35/42 (83%)	21/26 (80%)	χ²=0.21	0.65
Birth timing	Preterm	7/42 (17%)	5/26 (19%)	χ²=0.21	0.65
Birth timing: subgroup	Extremely preterm (<28 weeks)	1/42 (5%)	2/26 (8%)	-	1
Birth timing: subgroup	Very preterm (28-32 weeks)	1/42 (2%)	2/26 (8%)	-	0.55
Birth timing: subgroup	Moderate to late preterm (32-32 weeks)	5/42 (12%)	1/26 (4%)	-	0.39
Obstetric outcome	Meconium-stained amniotic fluid	5/42 (12%)	3/26 (11%)	-	1
Obstetric outcome	Fetal condition (*)	5/42 (12%)	3/26 (11%)	-	1
Obstetric outcome	Eutocia	28/49 (57%)	16/31 (52%)	χ²=0.23	0.63
Obstetric outcome	Dystocia	14/49 (29%)	9/31 (29%)	χ²=0	1
Obstetric outcome	Instrumentation	9/49 (18%)	4/31 (13%)	χ²=0.42	0.52
Obstetric outcome	Cesarean	12/49 (24%)	12/31 (39%)	χ²=1.83	0.18
Neonatal outcome	Apgar ≤7 at 1 min	7/41 (17%)	10/25 (40%)	χ²=4.27	0.04
Neonatal outcome	Umbilical pH <7.2	9/37 (24%)	4/20 (20%)	-	0.75
Neonatal outcome	Neonatal intensive care unit	11/42 (26%)	16/26 (62%)	χ²=8.38	0.004
Neonatal outcome	Median days hospitalization (IQR)	4 (1-72)	6.5 (1-85)	U=389.0	0.04
Neonatal outcome	Fever	7/42 (16%)	14/26 (53%)	χ²=5.2	0.02
Neonatal outcome	Microorganisms	5/42 (10%)	9/26 (29%)	-	0.02

However, several neonatal outcomes improved during the pandemic period, including lower rates of low Apgar scores (17% vs. 40%; p=0.04), NICU admission (26% vs. 62%; p=0.004), shorter neonatal hospital stay (4 days vs. 6.5 days; p=0.04), and reduced neonatal fever (16% vs. 53%; p=0.02).

During the pandemic period, four women (8%) tested positive for SARS-CoV-2. One patient developed severe illness characterized by fever up to 39.2°C, a respiratory rate of 28 breaths per minute, and oxygen saturation of 89%. She was a 41-year-old primiparous woman with obesity (BMI >30 kg/m²) and was co-infected with *Escherichia coli *sepsis.

All SARS-CoV-2-positive patients delivered vaginally at term and initiated breastfeeding. None required ICU admission, and the length of hospital stay ranged from two to nine days. Newborns were asymptomatic and remained hospitalized in the maternity ward for 2-5 days. No neonatal testing for SARS-CoV-2 was performed, and no maternal or neonatal readmissions were reported within 40 days after delivery.

## Discussion

The comparison between the two periods showed that the etiology of fever during labor, the distribution of bacterial agents, and maternal adverse events were comparable. There was a higher rate of maternal peripartum fever during the outbreak than in the control cohort (3.2% vs. 2.1%; p=0.07), which may be explained by closer monitoring of clinical parameters during the COVID-19 period compared with the pre-pandemic period.

As expected, intra-amniotic infection was the most frequently identified cause in both cohorts (22% vs. 39%; p=0.12). The second most common cause described in the literature is endometritis [5], whereas in our study, it was urinary tract infection (16% vs. 13%; p=0.75). This finding could be attributed to the use of intrapartum antibiotic prophylaxis in both groups, which likely reduced the incidence of endometritis but not of urinary tract infection [16,17].

Four cases of pneumonia were observed during the pandemic period, while none occurred in the control group. All were COVID-19-negative, but 75% (3/4) were tested only once, and given that the sensitivity of nasopharyngeal PCR testing is approximately 89% [18], a false-negative result cannot be excluded. These cases may have represented COVID-19 manifestations, co-infections, or secondary bacterial infections in patients with previous SARS-CoV-2 infection [19].

Among bacterial agents, the incidence of *E. coli* infection during and outside the pandemic (20% vs. 32%; p=0.23) was higher than expected [20], whereas group B *Streptococcus *(GBS) infection was lower in both periods (8% vs. 6%; p=1) [21]. The implementation of our local intrapartum antibiotic prophylaxis protocol likely explains the reduction in GBS infections. Indeed, previous publications have reported up to an 80% decrease in GBS infection incidence [22] and a stabilization of *E. coli *infection [23] following this prophylaxis. In our institution, the intrapartum antibiotic protocol consists of penicillin administration in cases of GBS positivity and amoxicillin-clavulanic acid (Augmentin) in cases of intrapartum pyrexia. This protocol remained unchanged during the COVID-19 period. Similarly, a study conducted in Rio de Janeiro, Brazil, reported a significant decline in GBS colonization among pregnant women during the COVID-19 pandemic compared with the pre-pandemic period (13.8% vs. 5.3%; p=0.0001) [24]. This reduction, independent of sociodemographic factors, suggests that pandemic-related control measures may have indirectly influenced the prevalence and characteristics of GBS colonization.

One in four (25%) COVID-19-infected patients developed severe illness, representing a higher rate than that described in the literature [10]. The clinical profile of this patient (obesity, advanced maternal age, and sepsis) corresponds to known risk factors for severe disease [25]. Although publications have reported increased maternal and neonatal complications among COVID-19-infected patients, we did not observe such findings in our cohort. However, the small sample size must be taken into account. The reported rate of neonatal infection ranges from 0% to 11.5% [26]. Unfortunately, in our study, no newborns were tested which limits comparison with previously published neonatal infection rates, although none presented symptoms consistent with infection. All mothers delivered vaginally and initiated breastfeeding [27].

Among the population studied during the outbreak, a significant improvement in neonatal outcomes was observed, including reduced NICU admissions (p=0.004), neonatal fever (p=0.02), and low Apgar scores (p=0.04). A positive impact of lockdown on birthweight has also been described in the literature, possibly related to changes in lifestyle and healthcare practices during lockdown resulting from reduced physical activity, decreased work-related stress, and greater attention to hygiene [28]. However, this finding has not been consistent across studies, and according to a meta-analysis published in 2021, there is insufficient evidence to conclude that lockdown measures significantly affected perinatal outcomes [29].

The main maternal complication of intrapartum pyrexia (cesarean delivery) was observed in both cohorts (24% vs. 39%; p=0.18) [6]. We also observed fewer cases of spontaneous rupture of membranes lasting more than 18 hours in the outbreak group (p=0.06). The shorter hospital stay observed during the outbreak period (p=0.045) likely reflects sanitary measures implemented during the pandemic.

To date, this study represents the first comparative analysis of peripartum fever conducted both during and outside the COVID-19 pandemic. Naturally, the limited number of cases in each group and the monocentric design restrict the generalizability of our findings. Moreover, this research was conducted prior to the emergence of SARS-CoV-2 variants and the implementation of vaccination.

## Conclusions

This study suggests that the COVID-19 pandemic did not significantly affect the etiology of peripartum fever or the distribution of bacterial and viral pathogens. Maternal and perinatal outcomes were overall reassuring, highlighting the resilience and effective adaptation of the healthcare system during the COVID-19 lockdown.

Although the outcomes of pyretic women in labor did not significantly change during the pandemic, an improvement in neonatal outcomes was observed during the outbreak period. This finding raises the hypothesis that changes in healthcare organization and clinical management during the pandemic may have influenced maternal and neonatal outcomes more than the infection itself. Further studies with larger populations are needed to confirm and better understand these observations.
